# AutoEPG: Software for the Analysis of Electrical Activity in the Microcircuit Underpinning Feeding Behaviour of *Caenorhabditis elegans*

**DOI:** 10.1371/journal.pone.0008482

**Published:** 2009-12-29

**Authors:** James Dillon, Ioannis Andrianakis, Kate Bull, Steve Glautier, Vincent O'Connor, Lindy Holden-Dye, Christopher James

**Affiliations:** 1 School of Biological Sciences, Bassett Crescent East, University of Southampton, Southampton, United Kingdom; 2 Signal Processing and Control Group, Institute of Sound and Vibration Research, University of Southampton, Southampton, United Kingdom; 3 School of Psychology, University of Southampton, Southampton, United Kingdom; Mount Sinai School of Medicine, United States of America

## Abstract

**Background:**

The pharyngeal microcircuit of the nematode *Caenorhabditis elegans* serves as a model for analysing neural network activity and is amenable to electrophysiological recording techniques. One such technique is the electropharyngeogram (EPG) which has provided insight into the genetic basis of feeding behaviour, neurotransmission and muscle excitability. However, the detailed manual analysis of the digital recordings necessary to identify subtle differences in activity that reflect modulatory changes within the underlying network is time consuming and low throughput. To address this we have developed an automated system for the high-throughput and discrete analysis of EPG recordings (AutoEPG).

**Methodology/Principal Findings:**

AutoEPG employs a tailor made signal processing algorithm that automatically detects different features of the EPG signal including those that report on the relaxation and contraction of the muscle and neuronal activity. Manual verification of the detection algorithm has demonstrated AutoEPG is capable of very high levels of accuracy. We have further validated the software by analysing existing mutant strains with known pharyngeal phenotypes detectable by the EPG. In doing so, we have more precisely defined an evolutionarily conserved role for the calcium-dependent potassium channel, SLO-1, in modulating the rhythmic activity of neural networks.

**Conclusions/Significance:**

AutoEPG enables the consistent analysis of EPG recordings, significantly increases analysis throughput and allows the robust identification of subtle changes in the electrical activity of the pharyngeal nervous system. It is anticipated that AutoEPG will further add to the experimental tractability of the *C. elegans* pharynx as a model neural circuit.

## Introduction

The nematode *Caenorhabditis elegans* has been widely used as a model organism to study different aspects of neurobiology. The availability of a fully sequenced genome, a well defined nervous system and a repertoire of simple behaviours, make *C. elegans* a highly tractable system to improve our current understanding of the relationship between molecular function at the cellular, systems and behavioural level. Indeed *C. elegans* has provided for nearly 50 years novel insight into a number of genes that have mammalian homologues, which are involved in complex mammalian behaviours [1].

So far *C. elegans'* behaviour has predominantly been studied by direct observation. A major caveat of this is that subtle phenotypes, which are difficult to detect/measure by observation are potentially missed. Over recent years the behavioural analysis of *C. elegans* has become increasingly refined with the introduction of automated computer systems [2]. In general automated systems serve three main purposes, i) they reduce the time it would take to conduct the experiment by direct observation ii) they standardize behavioural features to allow consistent analysis and iii) they enable features of behaviour to be rigorously quantified from individuals or large populations for the identification of subtle phenotypes. In particular automated systems combined with statistical approaches have facilitated a more sophisticated analysis of *C. elegans* rhythmic behaviours, such as locomotion and swimming [3], [4], [5], [6], [7], [8], and enabled a more detailed study of the genes and circuitries that underpin them.

The *C. elegans* pharynx is established as a model system for neural networks, both because of its relative simplicity and its amenability to both behavioural and electrical analysis [9]. The pharynx is a neuromuscular organ located in the head of the worm. The synchronous contraction and relaxation of the pharyngeal muscle cells facilitates the uptake and processing of food. Laser ablation studies of the circuitry suggest that the feeding activity or ‘pumping’ of the pharynx is myogenic [10] and modulated by the pharyngeal nervous system, a circuit of 20 neurons that is connected to the rest of the nervous system via a single interneuron. Consistent with other neural networks it expresses a combination of both classical and non-classical (peptidergic) transmitters, making the *C. elegans* pharynx a good experimental model for other neural networks [11].

**Figure 1:**
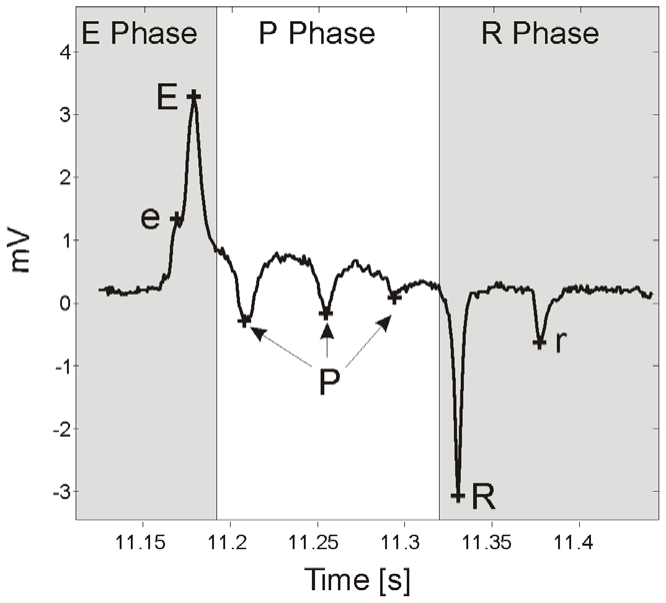
A typical EPG pump with annotated spikes. ‘e’ is the small excitatory spike (previously designated E1 [9]), caused by MC activation [12], [35]; ‘E’ is the large excitatory spike (previously designated E2 [9]), corresponding to rapid muscle contraction [12]; ‘P’ are the small negative spikes, caused by M3 activation [12]; ‘R’ is the large negative spike (previously designated R1 [9]), corresponding to rapid relaxation of the corpus muscle [9] and ‘r’ is the small negative spike (previously designated R2 [9]), corresponding to relaxation of the terminal bulb [9].

The electrical activity of the pharyngeal muscle during pumping can be measured extracellularly with a technique developed by David M. Raizen and Leon Avery [12], called the electropharyngeogram (EPG). Although pharyngeal behaviour can be measured by observation (i.e counting pumping), the EPG enables a more sophisticated analysis of pharyngeal behaviour and the underlying neuro-muscular circuitry. In an EPG recording a single pharyngeal pump produces a stereotypical waveform (Figure 1) which is composed of 5 distinct electrical transients. Each of these have been assigned their own annotation and occur in the following order (the EPG annotation that has previously been described is shown in brackets): e (E1) is a small positive spike corresponding to activity of the cholinergic motorneuron MC; E (E2) is an upwards spike that corresponds to muscle depolarization; P (P) is a plateau phase, where the muscle is depolarized and contracted and typically displays several negative spikes that are mediated by glutamate release from the M3 motorneuron; R (R1) is a large downwards spike and corresponds to the relaxation and repolarization of the corpus and r (R2) is a smaller downwards spike which corresponds to repolarization of the terminal bulb muscle. These electrical ‘landmarks’ of the EPG waveform have been used to extract and quantify discrete aspects of pharyngeal behaviour, such as pump rate [13] and pump duration [13], [14]. Quantification of such features can be used as a read-out for the efficacy of neuroactive drugs and to delineate effectors of signalling pathways, by the analysis of transgenic strains. For example, use of the EPG has made a significant contribution to our understanding of glutamatergic signalling pathways in the pharynx [12], [15], [16] and aided in the functional characterisation of several genes including those that have mammalian homologues like *eat-4*, the *C. elegans* vesicular glutamate transporter. This was possible because animals expressing putative null alleles of *eat-4* have a markedly different pharyngeal phenotype to wild-type, N2 [12], [16].

**Figure 2:**
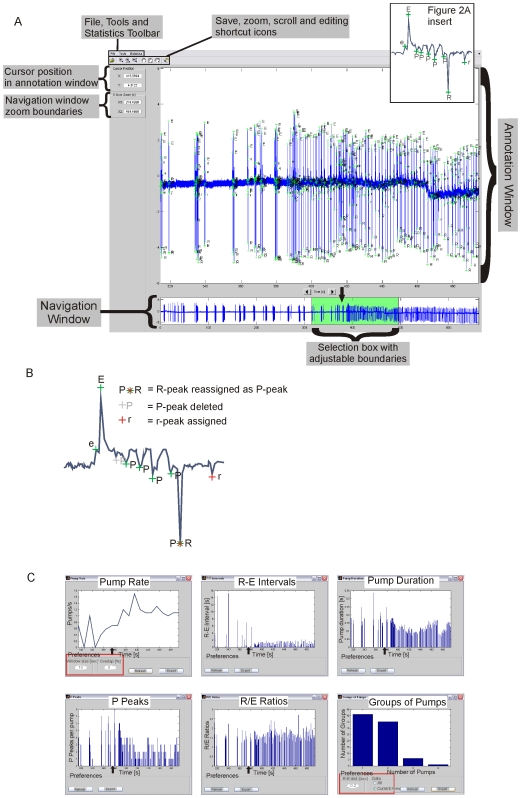
Overview of the AutoEPG system. A) A labeled screenshot of the AutoEPG graphical user interface. An example of an EPG recorded from a wild-type N2 worm is shown to demonstrate the capabilities of the interface. Briefly, after 6.2 min of recording in Dent's saline 100nM 5-HT, a potent stimulator of pharyngeal pumping, was applied. The entire recording is displayed in the navigation window and the time point at which 5-HT was applied is indicated by the black arrow (this was added retrospectively). The region of the trace consisting of 1 min immediately prior to 5-HT application and 2 min after application is easily selected in the navigation window (highlighted in green by AutoEPG). This selected region is displayed in the annotation window, where the algorithm annotation of the recording can be viewed. The highlighted region selected can be altered by simply clicking and dragging the left and right edges of the green box in the navigation window, or by clicking on the highlighting box and dragging it left or right with the mouse. Figure 2A Insert. An EPG waveform as it would appear having been annotated by the AutoEPG algorithm B) A cartoon illustrating the editing functions available in AutoEPG. C) Screenshots of the statistics that can be performed using AutoEPG. The pump rate, R-E interval, pump duration, P-peaks/pump and R/E-ratio/pump are displayed for the region of the recording selected in the navigation window in Figure 2A. The default of the statistics functions is to display data for the entire recording. However, when a region is selected in the navigation window the statistics function updates itself to display the statistics for the region of interest. In each case the black arrow indicates the time point at which 100nM 5-HT was applied (this has been added retrospectively). The groups of pumps statistic was performed on the first 2 minutes of the 5-HT application. The user-modifiable preferences of the statistics pump-rate and groups of pumps are outlined in red. In the case of pump duration, P-peaks and R/E-ratios each bar in the graphical output represents a single pump, with time on the x-axis. In the case of pump rate the user-modifiable preference ‘Window size (sec)’ refers to the time base used to plot the instantaneous rate, i.e if the size of window is set to 10 seconds as in this example, the pumps/sec will be calculated for each consecutive 10 sec ‘window’ of the trace. In the case of groups of pumps in this example the time interval has been set to 200 msec (i.e. consecutive pumps that occur within 200 msec of each other will be classified by AutoEPG as belonging to the same group). The analysis can be performed on the ‘Current View’ which is the region selected in the navigation window or on the entire trace by selecting ‘All.’

Although the EPG is relatively easy to conduct, its usefulness is limited by the time it takes to analyse the traces. Currently this is most accurately performed manually by cursor measurements on the digitized traces and although previous reports have quantified EPG recordings using third party software, they appear to offer only a narrow range of analysis capabilities [13], [17]. In view of this, we have developed an EPG analysis toolbox (AutoEPG) that offers a graphical user interface driven system for the automated annotation and statistical analysis of whole EPG traces (see Figure 2 and *Methods* for a detailed description) enabling the quantification of subtle pharyngeal phenotypes. To test the effectiveness of AutoEPG we have conducted a series of validation steps. Firstly we have shown that the AutoEPG algorithm is capable of analyzing large volumes of data (∼2500 single pumps were analysed in the case of one strain) with high levels of accuracy, based on the comparison of manually corrected data. Secondly we have analysed *eat-4(ky5)* animals which have pharyngeal phenotypes that can be resolved in greater detail with the EPG [12], [16]. In doing so we have quantified these pharyngeal phenotypes and demonstrated AutoEPG is capable of identifying the same features as those previously reported. In addition we have analysed EPGs recorded from SLO channel mutants. SLO channels are calcium-dependent potassium ion channels and have a role in patterning neural activity in the mammalian nervous system [18], [19], [20]. Using AutoEPG we have identified and quantified previously uncharacterised pharyngeal phenotypes of the null mutant *slo-1(js379)*. We have shown that *slo-1(js379)* has an altered pattern of pharyngeal pumping, which was rescued by specifically expressing the splice variant *slo-1_a_* in neurons. Moreover we have been able to show that the shape of the EPG waveform is altered by loss of *slo-1* function in ways that have not previously been described, further highlighting the utility of AutoEPG for the rapid identification of pharyngeal phenotypes.

## Materials and Methods

### Culturing of *C. elegans*

*C. elegans* strains were cultured following standard methods [21]. Hermaphrodite animals were fed and grown on a bacterial lawn (*E. coli*, OP50 strain) and the age of experimental animals was synchronized by picking L4 larval stage animals to new plates 24 hours prior to experiments being performed. Wild-type Bristol N2, *eat-4(ky5)* and *slo-1(js379)* strains were obtained from the *Caenorhabditis* Genetics Centre.

### Rescue Constructs

Plasmid pBK3.1 expressing the coding region of *slo-1_a_* under the control of the *snb-1* promoter, driving expression of *slo-1_a_* pan-neuronally, was kindly provided by Lawrence Salkoff [22]. A second construct to drive expression of *slo-1_a_* in the pharyngeal muscle was constructed by ligating the *slo-1*_a_ sequence from pBK3.1 downstream of the *myo-2* promoter sequence in plasmid pPD30.69 (a gift from Andrew Fire). Animals carrying the putative *slo-1* null allele, *js379* [22] were transformed by microinjection with these constructs. pPD118.33, which results in the expression of GFP in the pharyngeal muscle, was used as a co-injection marker to select transformed animals.

### EPG Recordings

Individual, well fed, one day old adult hermaphrodites were transferred to a 3cm diameter petri dish containing modified Dent's Saline (in mM: 10 D-Glucose, 140 NaCl, 1 MgCl_2_, 3 CaCl_2_, 6 KCl, 10 HEPES; pH 7.4) supplemented with 0.01% BSA w/v. A razor blade was then used to make a transverse cut immediately posterior of the terminal bulb of the pharynx. The semi-intact pharyngeal preparation containing the nerve ring and pharynx was transferred to the recording chamber where recordings were made at 20–22°C.

EPG recordings were made using previously described methods [23]. Briefly, a suction pipette (pulled from 1mm diameter borosilicate glass capillary to give a tip diameter of 12–15µm) was back-filled with Dent's Saline from the recording chamber and placed adjacent to the anterior region of the preparation, whereupon suction was then applied to attach the preparation. The reference electrode was a silver chloride-coated silver pellet in 3M KCl connected to the recording chamber by an agar bridge. Extracellular voltage recordings were made in “bridge” mode using an Axoclamp 2B amplifier (Axon Instruments) connected to a Digidata Box (Axon Instruments). The extracellular potential was set at 0mV, using the voltage off-set immediately prior to recording. Recordings were made in the presence of perfusion at a constant rate of 4 ml/min. Data were acquired using Axoscope (Axon Instruments) and recorded with a sampling rate of 2 kHz. The background noise of the setup was measured from the digital recordings and was typically 200–300µV. To minimise potential variation between recordings from individuals and strains, experiments were performed on animals that had been staged as L4 larvae and allowed to mature to young adults overnight. The recording conditions were standardized by checking the positioning of the recording electrode and recordings only showing ≤300µV noise and <100µVmin^−1^ baseline drift were used.

### EPG Model and Assumptions

In the development of the algorithm we made a number of assumptions about the shape of the EPG, which were based on the known range of EPG parameters from prior manual analyses and helped constrain the detection problem. The first assumption was that the distance between the E and R spikes (or the duration of the pump) cannot be greater than 1 second or smaller than 20 msec. Additionally, the distance between the r and R spikes cannot be greater than 1 second and the distance between e and E should be smaller than 200 msec. The most prominent feature of an EPG pump is the relaxation spike R, and therefore the algorithm identifies these features first. Subsequently, the algorithm looks for the E spikes and once these are found, the EPG trace is segmented into pumps. In the final stage, the smaller features (P, e, r) are sought and defined for each detected pump.

Central to the development of the algorithm was the modeling of the background noise, which is present in most EPG recordings. The noise can have two main components: a slow baseline drift (typically ∼60 µVmin^−1^) and a high frequency component (mostly attributed to the imperfect seal between the worm and the pipette). Although both types of noise can be removed in a preprocessing stage by a cascade of low and high pass filters, the filtering itself can introduce artifacts that distort the shape of the pumps. Instead of explicitly filtering the background noise we have designed our algorithm to tolerate a certain amount of background noise, which should be nevertheless low, assuming a good quality recording. The effect of the baseline drift was compensated by making the detection of the spikes independent of their absolute value. Although the detection of the major features of a pump (E and R spikes) is not affected by the high frequency noise, the detection of the lesser features (P, e, r) is more vulnerable to it. To counter the effect of this type of noise, its power was first estimated and all the subsequent detection thresholds were made dependent on its value.

### Detection of R Spikes

**Figure 3:**
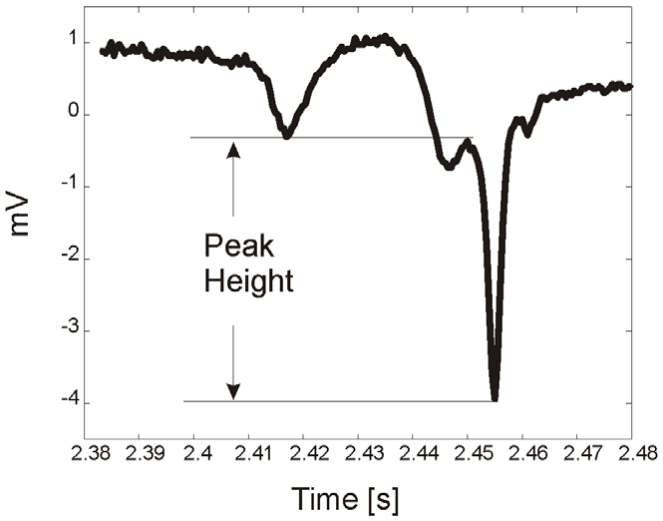
An R spike indicating how the amplitude measurement was made.

Traditionally the detection of spikes in biomedical signals is performed using information from the derivatives of a signal [24]. In this work for analysis of EPG spikes, we supplement this information with data from the height of each EPG spike. We defined the height of each peak as the distance between its tip and the nearest point where its first derivative changes sign (see Figure 3). For our convention we established that crests have positive height and troughs negative. The detection of R spikes was performed by finding all the peaks of the signal first and then calculating their height. The standard deviation of the heights of all the peaks was then calculated and those troughs that had a height more than 8 times the standard deviation were considered as R spike candidates.

We found that some of the R spike candidates could actually be sharp P spikes, for which the tip was higher than that of the tip of the R spike that belongs to the same pump. These misclassified R spikes were filtered out using two further tests. Firstly, a window of 1 second around the candidate R spike was selected and its median value was found. If the distance between the median and the candidate R peak was larger than the distance between the candidate and the lower peak contained in the window (potentially a neighbouring R spike) the candidate passed the test and if not it was discarded. Secondly the distance between consecutive R spikes was checked. The peak with the highest value was discarded if the distance was less than 0.02 seconds.

### Detection of E Spikes

Consistent with previously described EPG analysis [9] we found that every R spike had a corresponding E spike. We established that the candidate for the 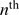 spike 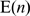 was the sample with the highest value within a window that precedes the 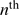 spike 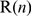. The size of the window was either equal to the distance between 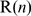 and 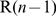 spikes, or the maximum pump duration assumed by our model, which was set to 1 second.

To achieve consistency with the model requirements about the minimum duration of a pump we checked the distances between all the pairs of E and R spikes. If the distance between 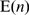 and 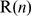 was less than 0.02 seconds, we merged the 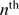 pump with one of its neighbours. In the merging procedure we assumed that the E spike was the highest point of a pump, while the R spike was its lowest. According to this, if the distance between 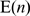 and 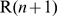 was less than 1 second and 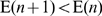 and 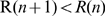, then 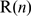 and 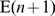 were discarded and 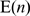 and 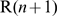 were assigned to the same pump. If any of the above conditions failed, a merging of the 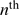 with the 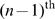 pump was then attempted based on the same principles. If both of these attempts failed then 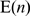 and 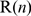 were discarded.

### Detection of P Spikes

The accurate detection of P spikes is less straightforward because the number of P-spikes in each pump is variable and their amplitude can be comparable to the level of the background noise. The strategy for the detection of P spikes comprised of three main steps. First the background noise was estimated and this was subsequently used in the development of noise adaptive thresholds. Second, we applied smoothing filters suitable for extracting peaks that are wide and shallow or peaks which are well defined but small in amplitude. Finally we calculated a score for each candidate P spike, which was based both on the height of the peak as well as on its position relative to the base line of the P phase plateau. The rationale behind using this dual metric was so that a) a deep peak registered as a P spike regardless of its position relative to the pump and b) P spikes that are small but sufficiently far from the P phase plateau were recognised.

The estimation of the background noise level was performed using the first order recursion algorithm (outlined below). For the 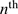 sample, the estimates for the mean and standard deviation were respectively 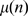 and 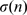. 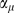 is the smoothing parameter for the estimation of the mean, which was set to 0.8/Fs, where Fs is the sampling frequency of the EPG. The respective smoothing parameter for the standard deviation estimate, 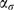, was set to 0.1/Fs. 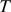 is a sensitivity factor that has experimentally been set to 4. We assumed that the power of the noise does not change for the duration of the EPG recording, therefore we established an overall estimate of the noise standard deviation as 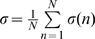. This was used for adapting the thresholds used for the detection of the P spikes (see below).
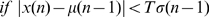


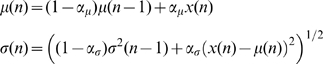

*else*

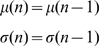

*End*

**Figure 4:**
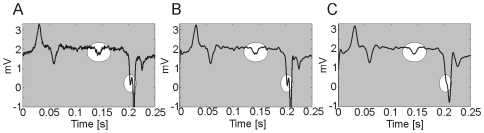
A pump filtered using two filters with different cut-off frequencies. A. The raw signal (unfiltered). B. The signal after being filtered with the 200 Hz cut-off filter. C. The signal after being filtered with the 100 Hz cut-off filter.

In order to remove some of the random variability due to the noise while preserving the overall shape of the P spikes, we filtered each pump with two low pass Butterworth filters of 4^th^ order. The filter with the lower cut-off frequency (100 Hz) targets the wider P spikes, while the second filter with a 200 Hz cut-off was designed to preserve the smaller spikes. Figure 4 shows the effect of the two filters on a pump that has both a wide P spike that is rather noisy, and a small P spike just before the R. Note that the filter with the higher cut off frequency captures the small spike, but unlike its lower frequency counterpart does not smooth the wide P spike sufficiently so as to facilitate its detection.

After filtering, the peaks of each pump's P plateau and their respective heights were extracted using the same algorithm used for the R spikes. Upon identification of the peaks we established two scores according to the height or the position of each peak. The height score 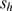 was defined as the ratio of a peak's height 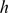 divided by 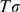, i.e.,
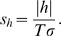
For the position score 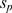, we first estimated the average level of the P plateau 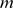, which was calculated as the mean of the 50% of the samples with the higher value that belong to the plateau. The position score was then defined by the ratio of the difference between 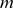 and the tip of the peak 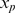, divided by 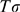, i.e.,
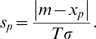
Finally, a peak was classified as a P spike when the product of the two scores was greater than 1, i.e. 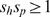.

### Detection of the e and r Spikes

**Figure 5:**
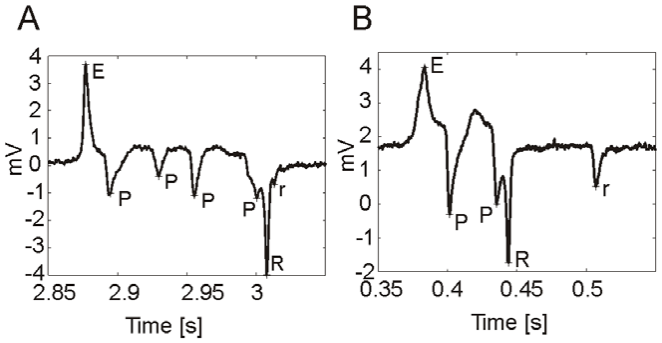
Two EPG pumps recording from wild-type, N2, showing the different temporal positioning of the ‘r’ spike within the EPG waveform. A. In this example the temporal distance between the R and r spike is small, thus the r spike is not distinct but instead appears as a small deflection upon the rising slope of the R-spike. B. In this example the temporal distance between R and r spikes is greater and so the r spike appears completely detached from the R spike.

The detection of the e and r spikes was performed essentially with the same algorithm used for the P spikes, with some minor adaptations that arise from the different nature of these features. Compared to the P spikes, e and r spikes are generally narrower and can either be detached from their companion E and R spikes, or they can be so close that they appear as a small deflection on the slope of the main E and R spikes. An example for the r spike is shown in Figure 5.

Similar to the detection of P spikes a smoothing filter was applied. As the e and r spikes are generally very narrow the 200 Hz cut-off filter and the raw signal were used. This was because e and r spikes can be very small and sensitive to filtering. The peaks and their respective heights were calculated using the algorithm applied for the detection of the R spikes. The height and position scores 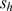, 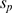 were extracted with the same method used for the P peaks. The only difference was that the average level 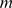 of the searching window was now the median value of its samples. Finally, the peak with the higher product 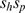 was registered as the e or r spike. A spike was not registered if the peak had a product less than 1.

### Definition of R-Spike and E-Spike Amplitude Used to Measure the R/E-Spike Ratio

The R-spike and E-spike amplitude for a single pump was calculated relative to the median value of the baseline following the pump. The region immediately following the R-spike, either until the following E-spike belonging to the next pump or for 1 second after the R-spike (which ever was shorter) was defined as the baseline. The amplitude of the E and R-spikes was then found by calculating the height distance between the E or R-spike and the median value of the baseline. The ratio of the two was then described as the R/E ratio for the pump.

### Overview of the AutoEPG System

The AutoEPG graphical user interface (GUI) is composed of two windows, the annotation and navigation windows (Figure 2A), which have different capabilities. When an EPG trace is first opened in AutoEPG the entire trace is displayed in the annotation and navigation window. By selecting ‘Analyse’ from the ‘Tools’ submenu the recording is automatically analysed by the algorithm. The algorithm annotation is superimposed upon the EPG recording, where each individual peak recognized by the algorithm is labeled as either an e, E, P, R or r peak (Figure 2A insert) according to the criteria for identifying each peak encoded by the algorithm (described above). The annotated recording appears in the annotation window and the user can then manually inspect the algorithm annotated trace using the scroll and zooming functions located on the toolbar directly above the annotation window. Using these functions of the annotation window the user can make a detailed examination of the annotation generated by the algorithm and where necessary edit this annotation, using the editing function and then save it. This is so that alternative interpretations of ambiguous segments of the recordings can be facilitated. The editing function is designed in such a way that all editorial changes can be easily visualised by the user, both at the time of making the change and when the edited annotation is re-opened at a later date within the annotation window. Indeed points of editing are superimposed upon the automated annotation. Editing can be subdivided into three categories, deletion, reassignment and assignment. When a peak is incorrectly annotated the label can either be deleted, in which case it now appears grey or reassigned, in which case the new label is shown to the left of the old label together with a × to signify the deletion and reassignment of the peak. If the algorithm has failed to identify a peak, the user can manually label the peak, it will then appear with the new label and a + (Figure 2B). The editing function also has an inbuilt ‘fail-safe’ for editing errors made by the user, which do not conform to the criteria for peak assignment. For instance in the example shown in Figure 2B the R-peak of the EPG waveform has been reassigned as a P-peak and since an EPG waveform must contain an R-peak AutoEPG would recognise this error made by the user during editing and a prompt will appear when the user attempts to perform the statistics. The GUI informs the user of the error that has been made and the time point within the trace at which it occurs; until this error is corrected statistics cannot be extracted from the recording.

In essence the corresponding region of the trace being displayed in the annotation window is highlighted in green in the navigation window. The navigation window therefore serves two key functions: i) it enables the user to maintain their bearings within the context of the whole trace whilst zoomed in upon a particular portion within the annotation window ii) it can be used to select specific ‘portions’ of the trace for analysis. For example, in Figure 2A the recording contains a pharmacological intervention; 100nM of 5-HT, a potent stimulator of pharyngeal network activity, has been applied to the pharynx at 6.2 minutes into the recording. The navigation window has been used to literally ‘highlight’ (as if with a highlighter pen) a specific time period, corresponding to 1 min directly before and 2 min after 5-HT has been applied, this same region is displayed in the annotation window. The X axis-zoom [s] function, displayed to the left of the annotation window displays the lower (X1) and upper (X2) time points of the region highlighted. This enables accurate adjustment of the boundaries of the highlighted region by the user. It is then possible to perform certain statistical analysis on this region in isolation from the adjacent portions of the trace, which are not highlighted. This is of particular use if the recording contains several pharmacological interventions for example during a dose-response analysis, where increasing concentrations are being applied during a single recording.

Once the user is satisfied with the signal peak annotation of the EPG recording, AutoEPG can be used to extract 6 statistical features, which are as follows: the duration of each individual waveform/pump; the instantaneous pump rate; the R-E interval, which is the time interval between consecutive individual pumps; the number of P-spikes per pump; the E-R ratio, which is the amplitude of the E-spike relative to the amplitude of the R-spike and the clustering together of pumps into groups. When a particular statistical analysis is performed a new window opens, in which a graphical display of the result is presented (Figure 2C). Within this window certain user-modifiable parameters are available, depending on the preference of the user (Figure 2C), which are as follows. The time frame used to measure the instantaneous rate can be adjusted in seconds. For example when the user wishes to look at a rapid rate change (seconds) a short time base (10s of seconds) may be more suitable to highlight this, or alternatively a slow change (several minutes) may be better portrayed by a longer time base (100s of seconds). In addition the overlap between consecutive time frames can be adjusted (0–99%), we found that 0% overlap enabled sharp changes in activity (such as when a drug is applied) to be better visualized than when an overlap is used (see Figure 2C). The second of the statistical analysis that can be altered is ‘Groups of Pumps’. Pump groups refers to the organization of individual pumps into clusters or groups. An individual pump is defined as belonging to a group if it occurs within a certain time distance (msecs) from the previous pump. This time distance can be defined by the user using AutoEPG, which then extracts the number of each group size, i.e the number of groups containing 1, 2, 3, 4 etc. individual pumps that conform to the criteria set by the user with the preference facility. The ‘Current View’ feature of the groups of pumps statistic can be combined with the navigation window to measure pump clustering/bursting within specific regions of the recording highlighted in the navigation window. For example in Figure 2C the groups of pumps have been measured in the 2 minutes directly after 5-HT application.

While a basic statistical analysis of the above information is available within the scope of the developed software, its exportation is also possible for further processing using data analysis packages (e.g. Microsoft Excel, SPSS and other statistics packages). As previously mentioned a new window is opened and a graphical presentation of the statistic being analysed is shown. From this window the user can export the data to a statistical package, which will allow it to be manipulated. When the data is extracted, it appears in Excel together with the parameter it corresponds to (i.e. pump duration etc…) and the source of the data, which is typically the name of the .abf/.atf file. This enables the user to accurately maintain the data and refer back to its source if necessary at a later date. The default extraction protocol of AutoEPG is to extract the data for the entire trace, once this has been done the user can select data regions that are of interest within the analysis package.

### Requirements for Running AutoEPG

AutoEPG is written in MATLAB® and distributed using a run time compiler freely available from The Mathworks (MATLAB®) and AutoEPG can be run on most Windows based PCs (tested on both Windows XP and Vista). An evaluation copy of AutoEPG can be downloaded for free in one click after reading and agreeing to the terms and conditions for acquiring this software from http://www.soton.ac.uk/~jamescj/EMbody-Biosignals/our-products.html. In the evaluation copy of AutoEPG certain functions have been disabled, which are as follows: statistics cannot be exported, only the first minute of any trace loaded can be analysed and the statistics are not updated when a region is selected by the navigation window (with the exception of the Groups of Pumps statistic). The full software is available free for academic research under an end user agreement, contact Embody-Biosignals for further details. AutoEPG is designed to analyse digital recordings stored in the .abf (axon binary file) and .atf (axon text file) file formats. AutoEPG is capable of accepting data recorded using other instruments, providing that it can be exported as a text file and we would be happy to accommodate other file types as the need arises. An example EPG trace is provided at the download site (denoted: Figure 2 Demo Trace) for those wishing to trial the software but do not have access to such digital recordings.

### Manual Correction of AutoEPG Annotation

With the exception of Table 1, data was obtained from EPGs that had been analysed using AutoEPG and undergone minimal or no manual correction. To clarify, the algorithm annotation of P, e and r spikes were not manually corrected unless a pump (i.e. an E-R spike pair) with which they were associated had been incorrectly identified. AutoEPG was used to perform statistical analysis of the EPG recording and in doing so the recording was ‘screened’ for anomalous pumps. If the anomalous pump correlated with an incorrect peak assignment the annotation was corrected using the editing features of AutoEPG.

**Table 1:** Validation of the accuracy and efficiency of AutoEPG at detecting the different electrical transients of the wild-type N2 EPG.

	N2	
	FNR (%)	Precision (%)
Pumps	0.4	100
e	2.1	94.9
r	2.1	82.5
P	1.0	99.6

The FNR. and Precision of the AutoEPG algorithm in detecting: pumps (corresponding to E- R spike pairs), e, r and P spikes. *N* = 5 worms and in total 271 individual pumps were analysed.

### Statistics

Statistical significance was determined using a two-tailed unpaired Student's *t*-test (statistical significance was set at *P*<0.05). The time period of the trace, the number of corresponding pumps and the number of individuals used to perform the statistical analysis for each strain is stated in individual figure legends.

## Results

### Validation of the AutoEPG Algorithm

Prior to using the EPG analysis tool to compare experimental data it was necessary to *i)* assess its accuracy in detecting single pumps (i.e. pairs of E-R spikes) and *ii)* check how accurately the software annotated the different spikes of the EPG waveform (e, E, R, r, P). This was done by performing an automated analysis of EPG traces using the software followed by the correction of any mistakes by hand. A comparison was then made between the hand corrected annotation and the automated one. When making this comparison the following definitions were employed; *False negative:* when the software *failed* to identify a spike; *False positive:* when the software identified a spike *incorrectly*; *True positive:* when the software identified a spike *correctly*. These parameters were used to define the following two measures of accuracy: the False Negative Rate (FNR) and Precision *(see below)*.
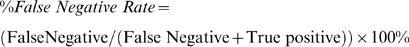


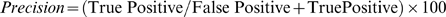



The FNR represents how accurate the automated analysis is at identifying a spike compared to the hand corrected annotation. A FNR of 0% indicates the software has successfully identified all of the same spikes as the manual correction, without missing any. Hence the lower the FNR the greater the accuracy is of the automated analysis. The ‘Precision’ represents how accurate the software is once it has identified a spike. A ‘Precision’ of 100% indicates that the software has not identified any additional spikes to those identified manually; hence a high precision value indicates a high accuracy.

The evaluation was performed on the first minute of EPG traces recorded from five individual wild-type animals under basal conditions. A total of 271 individual pumps were analysed (see Table 1). The FNR for the identification of pumps (E-R pairs) and P spikes was extremely low (0.4% and 1% respectively) and only slightly higher for the identification of the smaller amplitude e and r spikes (2.1%). The ‘precision’ for the identification of r spikes was 82.5% whereas pumps, e and P spikes were detected with a precision of ≥94.9%, demonstrating that AutoEPG is an accurate tool for the identification of EPG spikes and their correct annotation.

### Analysis of Mutant Strains with Known Pharyngeal Phenotypes

In order to demonstrate the utility of AutoEPG we used it to extract features from EPG recordings made from two different mutant strains. All data presented henceforth has been obtained from EPG recordings that have undergone minimal or no manual correction (see *Methods*). Compared to EPG recordings made from N2 animals the level of manual correction performed was similar for both mutant strains tested. In the first instance we sought to establish the software's ability to annotate an EPG waveform that has a significantly different profile to N2 and validate the software by comparing our findings to those previously published. The *eat-4(ky-5)* null mutant has a well defined pharyngeal phenotype [12], [16]. The gene *eat-4* encodes the glutamate vesicular transporter and *eat-4(ky5)* has defective glutamatergic neuro-transmission. This results in a loss of the motorneurone M3's glutamate mediated transmission. The major effect of M3 is to provide chloride-dependent hyperpolarization during muscle contraction which is mediated by post-synaptic muscle glutamate-gated anion channels. The *eat-4* mutation affects the shape of the EPGs recorded from these animals in two ways; they show fewer P-spikes per pump and an increased pump duration [12]. AutoEPG was used to analyse 5 minute EPG recordings made in Dent's saline from wild-type N2 (n = 16) and *eat-4(ky-5)* (n = 5) animals. In AutoEPG the pump duration is defined as the time interval between E and R spikes. Using this definition we have shown that the pump duration of *eat-4(ky5)* null animals is significantly longer (213.4±12.2 msec) compared to the wild-type (112.5±6 msec) (Table 2). We have also shown that there is a significant decrease in the average number of P spikes per pump in *eat-4(ky5)* animals (Table 2), which is consistent with previous publications. This indicates that AutoEPG is a reliable and efficient tool for the analysis of transgenic animals with pharyngeal phenotypes. It should be highlighted that in our experiments the EPGs were recorded under basal conditions, in the absence of chemicals or mutations that stimulate pumping. This would explain why the pump rate of *eat-4(ky5)* (Table 2) is lower than previously published values, which were obtained in an *unc-31* genetic background that constitutively pumps [16].

**Table 2:** A comparison of wild-type, N2 to *eat-4(ky5)* and *slo-1(js379)* pharyngeal behaviour.

Feature	N2 (N = 16)	*eat-4(ky5)* (N = 5)	*slo-1(js379)* (N = 21)
Pump Rate (Hz)	0.37±0.08	0.41±0.03	0.39±0.03
P-spikes/pump	2.5±0.15	0.161±0.05***	0.89±0.15***
Pump duration (msec)	112.5±6	213.4±12.22***	147±7**
R/E ratio	1.576±0.08	1.77±0.19	1.360±0.04*

All of the EPG features were extracted using AutoEPG to analyse the first 5 minutes of the recording in Dent's saline. For N2, *eat-4(ky5)* and *slo-1(js379)* approximately 1300, 600 and 2472 single pumps were analysed respectively, in total. (A two-tailed unpaired student's t-test was used to compare mutant strains to wild-type. Asterisks: *p<0.05, **p<0.01, ***p<0.001. Each value shown represents the mean±SEM).

### Quantitative Analysis of slo-1 Pharyngeal Phenotypes

Next AutoEPG was used to analyse worms with mutations in the *slo-1* gene that encodes a calcium-dependent potassium channel [22] and to which we had previously qualitatively ascribed a variation in its patterns of activity in the EPG (Kate Bull, unpublished observation). SLO-1 is expressed in multiple pharyngeal neurons, but not at detectable levels in the pharyngeal muscle [22], [25]. This widespread cellular expression pattern suggests SLO-1 performs an important role in regulating the activity of the pharyngeal network. In the mammalian nervous system SLO channels have been identified as providing a calcium-dependent means of regulating the duration of excitatory synaptic events, at both pre and postsynaptic terminals [26]. This property makes SLO channels ideally suited for the temporal coordination of neuronal firing, and consequently the patterning of outputs by neural circuits that underlie rhythmic behaviours [27].

EPGs were recorded from the putative null mutant *slo-1(js379)* (n = 21) in Dent's saline. The first five minutes of recording was analysed using AutoEPG, corresponding to the analysis of between 15 and 270 pumps per individual and several different features were extracted for comparison to the wild-type (see Table 2). The mean pump rate of *slo-1(js379)* (0.39±0.03 Hz) was not significantly different to the wild-type (0.37±0.08 Hz). This was surprising since it has previously been reported that the pump rate of *slo-1(js379)* is faster [25]. However, this previous report was observed in a gap junction mutant background that has an unsynchronized pattern of pharyngeal muscle contraction. Using AutoEPG we analysed specific features pertaining to the shape of the EPG waveform of *slo-1(js379)* mutants and compared them to the wild-type. We found a significant decrease in the average number of P-spikes per pump in EPGs recorded from *slo-1(js379)* animals and this was accompanied by a significantly longer pump duration compared to the wild-type (see Table 2). It has been shown that P-spikes are caused by the release of glutamate from the M3 motorneurons [12] and our results would suggest that M3 glutamatergic neurotransmission is disrupted in mutants that lack SLO-1 function.

In EPGs recorded from *slo-1(js379)* animals the amplitude of the E-spike was measured relative to the amplitude of the R-spike amplitude (the R-E Ratio). The amplitudes were expressed in this way as the EPG spikes are sensitive to small variabilities in the recording conditions, such as the positioning of the electrode and the seal with the worms cuticle. To normalize for this inherent variability and quantify if there is a change in R and/or E-spike amplitude, the ratio of the R-spike to the E-spike amplitude was measured and recorded for each individual pump (see *Methods* for a precise description of how the spike amplitude was defined). Using this feature of AutoEPG we have quantified the mean R/E-spike ratio of *slo-1(js379)* pumps (Table 2) and identified that it was significantly reduced (1.36±0.04 mV) compared to the wild-type (1.576±0.08 mV), suggesting that in *slo-1(js379)* animals normal muscle contraction and/or relaxation is impaired.

**Figure 6:**
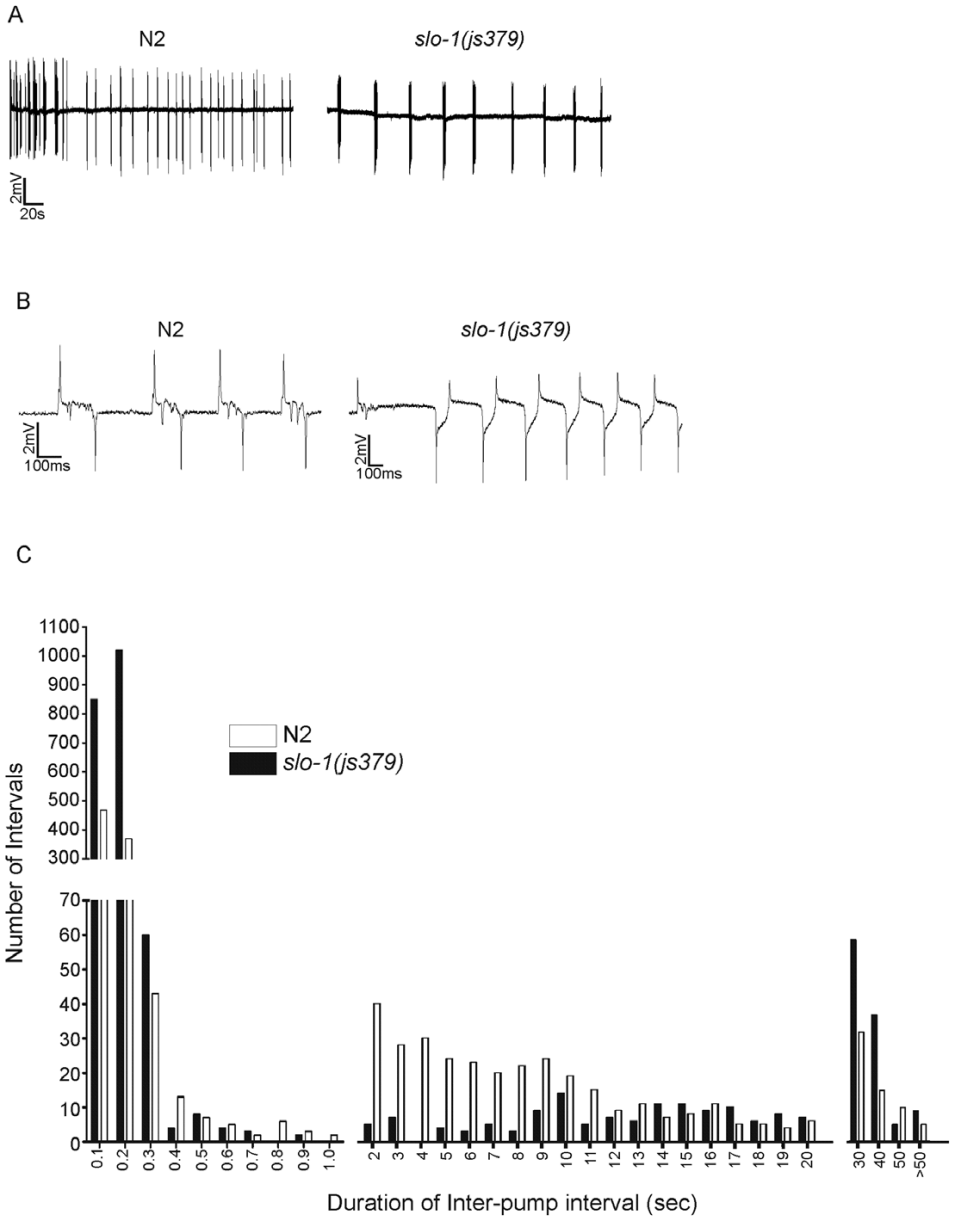
*slo-1(js379)* animals have an altered pattern of pharyngeal activity. A. Representative EPG recordings made from an N2 (wild-type) and *slo-1(js379)* worm perfused with saline at a rate of 4 ml/min within 2–3 min of the dissection. B. The time-base of the EPG recordings shown in A have been expanded so that individual pumps can be discerned, further highlighting the ‘bursting’ pattern of activity of *slo-1(js379)* animals. C. Inter-pump interval distribution of pumping activity in N2 and *slo-1(js379)* animals from 5 min of recording each. The intervals were divided into four groups and then further subdivided into smaller bins as follows: Up-to 1 second (100 msec); from 1–20 seconds (1 second); from 20–50 seconds (10 seconds) and >50 seconds. Note the high number of short intervals (0–300 msec) in *slo-1(js379)* compared to N2.

Although the pump rate of *slo-1(js379)* and wild-type animals was not significantly different, visual inspection of EPG recordings suggested that the mutant had an altered pattern of pumping and was prone to pumping in large ‘bursts’ of activity (see Figure 6A and B). To investigate this further AutoEPG was used to extract all of the inter-pump intervals for EPG traces recorded from *slo-1(js379)* and wild-type animals. The inter-pump interval was defined as the time between the R and E spike of consecutive pumps. The inter-pump intervals of *slo-1(js379)* and the wild-type were plotted as a frequency distribution (Figure 6C). The *slo-1(js379)* animals display a bimodal distribution, comprised of short (≤300 msec) and long (≥30 sec) durations, which would be expected considering the ‘bursting’ pattern of pumping. It also appeared that there was some inherent patterning of wild-type pharyngeal activity, which consisted of short (≤300 msec) and intermediate (between 2 and 11 sec) inter-pump intervals. Indicating wild-type animals tend to pump in ‘bursts’, but have shorter intervals between ‘bursts’ compared to *slo-1(js379)* animals.

Using AutoEPG we then measured the group organization of individual pumps. It was apparent from the frequency distribution of inter-pump intervals that *slo-1(js379)* exhibited more with durations≤200 msec, compared to wild-type. Hence, using AutoEPG an individual pump was defined as ‘belonging’ to a group if it occurred within 200ms of the previous pump. With this criterion we identified that in *slo-1(js379)* 71% of groups contain ≥4 individual pumps, whereas in the wild-type this figure was only 27%. This difference was significant (p<0.001, using a two-tailed unpaired student's t-test) and would suggest that *slo-1* function performs an important role in modulating the pattern of output of the pharyngeal network.

### Rescue of slo-1mutant Phenotypes with the Tissue Specific Expression of SLO-1_a_

**Figure 7:**
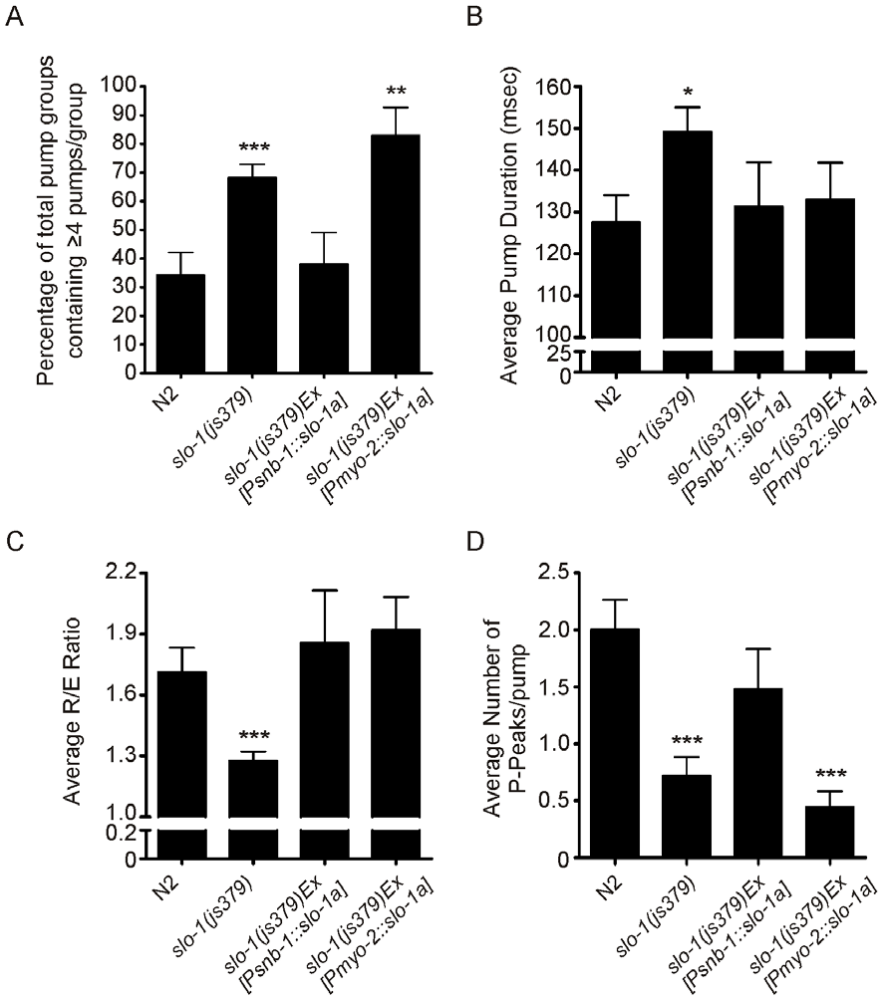
The rescue of *slo-1(js379)* pharyngeal phenotypes using either pharyngeal muscle or pan-neuronal specific rescue constructs. EPG recordings were made in Dent's saline from wild-type N2, *slo-1(js379)* and *slo-1(js379)* animals expressing *slo-1a* either in the pharyngeal muscle or in the pharyngeal nervous system. In this comparison the first minute of the recordings made from each strain were analysed using AutoEPG. The following features were measured: A. Pump groups; B. Average pump duration; C. Average R/E ratio and D. Average number of P-peaks/pump. The number of individuals' tested for each strain, together with the number of individual pumps (*P*) was as follows: N2, *N* = 16, *P* = 358; *slo-1(js379)*, *N* = 21, *P* = 556; *slo-1(js379)Ex[Psnb-1::slo-1a]*, *N* = 5, *P* = 90 and *slo-1(js379)Ex[Pmyo-2::slo-1a]*, *N* = 7, *P* = 232. (Two tailed, unpaired t-tests were used to compare transgenic lines to N2, wild-type; Asterisks: *p<0.05 **p<0.01, ***p<0.001; graphs show the mean±SEM).

As previously mentioned, *slo-1* has been identified as being expressed in the pharyngeal nervous system, but not in the pharyngeal muscle [13]. A number of different splice variants of *slo-1* exist in *C. elegans (slo-1_a–c_)*, of these *slo-1_a_* is the longest variant [13]. To test if the altered pharyngeal behaviour is a consequence of *slo-1* loss of function, *slo-1_a_* cDNA was expressed specifically in the neurons of *slo-1(js379)* animals, under the control of the synaptobrevin promoter (*Psnb-1::slo-1_a_*). In this comparison the first minute of recording was selected and analysed using AutoEPG, for each of the different strains. The pan-neuronal rescue of *slo-1_a_* function was sufficient to rescue the disrupted pattern of pharyngeal pumping (Figure 7A). The expression of *slo-1_a_* only in the pharyngeal muscle of *slo-1(js379)* mutants using the *myo-2* promoter (*Pmyo-2::slo-1_a_*) failed to rescue the ‘bursting’ activity of *slo-1(js379)* pharynxes consistent with the published expression pattern of *slo-1*. In addition we have used AutoEPG to determine if the other *slo-1* mutant pharyngeal phenotypes are rescued by the tissue specific expression of *slo-1_a_*. We found that the neuronal expression of *slo-1a* in *slo-1(js379)* mutants rescued all of the remaining pharyngeal phenotypes (pump duration, R/E ratio and P-peaks. Figure 7B–D). Whilst the expression of *slo-1_a_* in the pharyngeal muscle did not rescue the number of P-peaks/pump it did rescue the pump duration and the R/E ratio. Hence, this would suggest that SLO-1_a_ can function in the pharyngeal muscle despite there not being any evidence that it is expressed there.

## Discussion

The pharyngeal nervous system is a neural network which serves as a powerful model for the way in which microcircuits control complex behaviours. Accordingly, the genetic analysis of feeding behaviour has provided a pivotal route to defining key mediators at the core of evolutionarily conserved synaptic transmission mechanisms (*eat-4*) and distinct, invertebrate specific molecules that serve as important anthelmintic targets. The flexibility of this organ is re-iterated because a defined network of 20 neurons has a tangible read-out of a defined muscle pump that can be observed in the context of an intact organism or in an isolated *in-vitro* preparation. The electropharyngeogram enables the behaviour of the pharyngeal network to be monitored. However, the interpretation of the EPG read-out is currently limited by the lack of available analysis tools. Based on previous analysis the electrophysiological signature that the pharynx provides has shown its value in defining how environmentally driven behaviour can be mapped onto precise neurochemical behaviours [13], [28]. However, the quantitative comparison of this extracellularly derived signal has not been subjected to relatively established approaches that have proved useful in unpicking more complex electrophysiological data [29]. To address this we have developed AutoEPG, a semi-automated EPG analysis algorithm and graphical user interface that offers a broad range of capabilities and analysis features that are otherwise not available within a single package.

The key principle employed is peak-detection and noise deconvolution. The processes here have been established elsewhere and have been successfully used in fields such as epileptic spike detection [30]. Although relatively established, these principles when applied to the EPG analysis make for a robust and user friendly tool for defining the EPG in both a precise and accurate manner (Table 1). Moreover, AutoEPG allows the high throughput analysis of large volumes of data, decreasing analysis time by up to 1000 fold compared to previous manual investigation and would combine well with approaches for high throughput recording of EPGs. Thus, AutoEPG has the potential to be useful for the discovery and characterization of neurogenic or myogenic drugs. This would reinforce investigation of endogenously or heterologously expressed key drug targets including those that are emerging from mode of action studies of key anthelmintic drugs for which *C.elegans* is an important drug discovery/validation model. Additionally we have resolved to reduce variation while performing our background characterization by controlling the age and the experimental parameters. This has included using precisely staged animals and standardized recording conditions in which the positioning of the electrode on the pharynx was checked and recordings only showing ≤300 µV noise and <100 µVmin^−1^ baseline drift were used.

Here we have assessed the ability of AutoEPG to annotate the wild-type EPG, verified its use by investigating a well defined mutant and extended it to highlight its power at resolving previously ill-defined or uncharacterized mutants. We reasoned that this was an important validation step, since the EPG features quantified using the software are only as accurate as the annotation from which they are extracted. By comparing manually corrected and automated annotations we were able to show that AutoEPG is capable of high levels of accuracy, with respect to both detection of discrete signals and their correct annotation. We then analysed EPGs recorded from the mutant strain *eat-4(ky5)* and validated our findings against those previously published. With minimal or no manual correction AutoEPG successfully identified the same EPG phenotypes previously reported for *eat-4* mutant worms [12], [16], illustrating its ability to tolerate EPGs markedly different to the wild-type and reliably quantify them. In addition, we quantified other features of the *eat-4(ky5)* EPG such as R/E-spike ratio and pump rate. We found that under basal conditions these were not significantly different to wild-type, N2.

To further demonstrate the capabilities of the AutoEPG we analysed EPGs recorded from the SLO-1 loss of function mutant *slo-1(js379)*. We have identified that the morphology of the *slo-1(js379)* EPG waveform is distinct from wild-type in several ways. It has been shown that transgenic animals with M3 motorneurons ablated and deficient in glutamate transmission exhibit both increased pump duration and a complete loss of P-spikes [7], [9], [10]. We identified that *slo-1(js379)* animals have increased pump duration and fewer P-spikes. This would suggest that glutamatergic transmission in *slo-1(js379)* is reduced. It would be predicted that a loss of SLO-1 function in M3s would lead to an increase in glutamatergic transmission; however we do not observe this. Instead we suggest if SLO-1 regulates the activity of M3s, it is likely that this occurs within the network upstream of the M3 motorneurons. In *slo-1(js379)* animals we quantified a decrease in the R/E-spike ratio. As *slo-1* has not been identified in the pharyngeal muscle [22], [25] we would suggest that this change is caused by the loss of SLO-1 function in the nervous system, which co-ordinates the activity of the muscle and could reflect a desynchronized contraction and/or relaxation of the different pharyngeal muscle cells.

EPGs recorded from *slo-1* null mutants have previously been reported. In one study it was suggested that the EPG waveform was grossly similar to wild-type, with perhaps a shorter duration [22], although this was not quantified. Another study suggested that the pump rate was increased [25]. This contrasts with our findings, however, our results are not directly comparable to those previously described. Here we have performed our analysis under basal conditions, when the pharyngeal system is in a resting state, whereas previous observations have been made either from worms in a different genetic background which causes an uncoupling of coordinated pharyngeal muscle contraction [25] or when the pharyngeal system is in a hyper-excited state stimulated by the application of 5-HT [22]. It is known that 5-HT is a potent stimulator of pharyngeal activity and acts on multiple effectors at both pre and post-synaptic sites at the neuromuscular junction [13], [17], [31], [32], [33]. This might mask those phenotypes we have identified under basal conditions.

Although the pumping rate of *slo-1(js379)* appeared to be slightly faster than the wild-type, it was not significantly different. With AutoEPG we identified that the temporal organization of *slo-1(js379)* pumping was significantly different to the wild-type; the inter-pump interval of *slo-1(js379)* had a different distribution to wild-type and *slo-1(js379)* exhibited a greater tendency to pump in large groups containing ≥4 individual pumps. This phenotype has not previously been quantified and was rescued by the pan-neuronal expression of *slo-1_a_* in *slo-1(js379)* animals but not by the expression of *slo-1_a_* in the pharyngeal muscle. It would suggest *slo-1_a_* performs an important role in determining the output of the pharyngeal nervous system and timing the onset of electrical activity in the muscle. Mammalian homologues of SLO-1 have been identified as regulating the firing activity of mammalian neural circuits [18], [19], [34]. Indeed, cortical neurons exhibit a bursting pattern of neuronal activity in the presence of the BK channel blocker iberiotoxin [20]. Here, we also see a bursting pattern of activity in muscle that is driven by a neural network lacking SLO-1 signalling. In the pharyngeal nervous system the cholinergic motorneuron MC stimulates pharyngeal pumping and serves as a neuronal ‘pacemaker.’ A loss of SLO-1 function might change the activity of the MC neuron, which is seen as a change in the pattern of pumping. The other pharyngeal phenotypes of *slo-1(js379)* that were identified using AutoEPG were all rescued by the neuronal expression of *slo-1_a_*.

The *myo-2::slo-1_a_* rescue construct generates an over expression of *slo-1_a_* in the pharyngeal muscle, a tissue that does not ordinarily express this protein and rescued the decreased R/E-spike ratio and the pump duration, but not the average number of P-peaks/pump or pattern of pumping. The P-spikes correspond to M3 neuronal innervations, hence it would be predicted that these would not be rescued by the expression of *slo-1_a_* in the muscle. Whilst the rescue of the pump duration and R/E-spike ratio suggests the exogenous expression of *slo-1_a_* in the pharyngeal muscle is sufficient to functionally restore the normal behavior of the muscle. As *slo-1* encodes a voltage-activated Ca^2+^-dependent potassium channel which is expressed in body wall muscle [22] and can function in an autonomous fashion, we suggest that exogenous over-expression in pharyngeal muscle can generate a functional channel that contributes to pharyngeal muscle excitability. The visually observed expression pattern of *myo-2::GFP* would suggest that the *myo-2* promoter drives expression ‘tightly’ in the pharyngeal muscle. However, we cannot rule out that there may be some low level ‘leaky’ expression below visual detection within the nervous system, leading to a rescue of some but not all of the defects.

In the present study we have demonstrated that AutoEPG provides a new tool for the identification and quantitative description of pharyngeal phenotypes. In doing so AutoEPG has provided further insight into the function of SLO-1 in the *C. elegans* pharynx and highlighted a functionally conserved role of SLO-1 in regulating the pattern of activity within neuronal circuits. Furthermore, AutoEPG will enable the standardized and consistent analysis of EPG traces, in a fast and accurate manner, which will facilitate the efficient statistical analysis of large EPG data sets opening the way for higher throughput analysis. An evaluation version of AutoEPG is available online by following the link http://www.soton.ac.uk/~jamescj/EMbody-Biosignals/our-products.html (see *Methods* for further details).
